# Attitudes and local ecological knowledge of experts fishermen in relation to conservation and bycatch of sea turtles (reptilia: testudines), Southern Bahia, Brazil

**DOI:** 10.1186/1746-4269-9-15

**Published:** 2013-03-01

**Authors:** Heitor de Oliveira Braga, Alexandre Schiavetti

**Affiliations:** 1 Programa de Pós-Graduação em Ecologia e Conservação da Biodiversidade, Universidade Estadual de Santa Cruz - UESC, Rodovia Jorge Amado km16, Ilhéus, Brazil; 2 Departamento de Ciências Agrárias e Ambientais (DCAA), Universidade Estadual de Santa Cruz – UESC, Rodovia Jorge Amado km16, Ilhéus, Bahia, Brazil

**Keywords:** Marine turtle, Marine conservation, Taboos

## Abstract

**Background:**

The use of ethnoecological tools to evaluate possible damage and loss of biodiversity related to the populations of species under some degree of threat may represent a first step towards integrating the political management of natural resources and conservation strategies. From this perspective, this study investigates fishermen’s ecological knowledge about sea turtles and attitudes towards the conservation and bycatch in Ilhéus, Southern Bahia, Brazil.

**Methods:**

Fishermen experts semi-structured interviews were performed using snowball sampling method. The interviews consisted of a series of questions relating to the fishermen’s profile, structure and work equipment, the local ecological knowledge of fishermen about sea turtles and bycatch, a projective test, attitudes towards turtle conservation and beliefs and taboos regarding turtles. Indicators for quantitative comparisons of respondents in terms of their broad knowledge and attitudes towards turtle conservation were created. Correlation analyses were made between indicators of knowledge and attitude as well as the relationship between education level and knowledge and attitudes.

**Results:**

Thirty experts were interviewed for the study. The local ecological knowledge and attitudes of fishermen towards the conservation of sea turtles were respectively medium (0.43) and moderate (0.69) according to experts (based on Likert scale and Cronbach’s Alpha). Potential areas of spawning were reported from Barra Grande to Una covering the entire coast of Ilhéus. Methods for identifying the animal, behavior, and popular names were described by fishermen. The most recent captures of turtles were attributed to fishing line, but according to the respondents, lobster nets and shrimp traps are more likely to capture turtles. Knowledge and attitudes were weakly inversely correlated (r = −0.38, p = 0.04), and the education level of the respondent showed a positive correlation with positive attitudes towards turtle conservation (H = 8.33; p = 0.04). Life history, habitat, specific and exogenous taboos, beliefs and the use of hawksbill turtle to make glasses and other handcrafts are also reported in the study.

**Conclusions:**

Monitoring of spawning areas, preservation of traditional practices, strategies to moderate the use of fishery resources and the local ecological knowledge/attitudes can provide data to improve the conservation practices and management of sea turtles.

## Background

Sea turtles are susceptible to damage through various interactions with humans, due to the fact that they are migratory and occupy distinct geographical areas according to their stage of life [1,2]. On a global scale, all species of sea turtles in Brazil are under some level of threat according to the International Union for the Conservation of Nature (IUCN) [3], including the green turtle (*Chelonia mydas*) and loggerhead turtle (*Caretta caretta*), which are threatened with extinction; the olive turtle (*Lepidochelys olivacea*), which is vulnerable to extinction; and the leatherback turtle (*Dermochelys coriacea*) and hawksbill turtles (*Eretmochelys imbricata*), which are critically endangered species.

An assessment of the conservation status of turtles in Brazil performed by the Chico Mendes Institute for Biodiversity Conservation (ICMBio)/TAMAR (Sea turtles) Project, reported the green turtle *C. mydas* as vulnerable (VU), *C. caretta* and *L. olivacea* as in danger (EM) and *E. imbricata* and *D. coriacea* as critically endangered (CR), which indicates reductions in the populations of these taxa in recent years on the Brazilian coast [4-8].

There are several threats that these animals face in the sea or on the beaches where they nest [9,10]. Human activities and impacts such as vehicular traffic on beaches, plastic pollution, contamination with oil, the spreading of pathogens, the accidental capture of turtles by fishermen, the gathering of eggs and females on beaches, global climate changes, collisions of turtles with boats and the dredging of harbours and channels can be cited as the primary causes of the declines in turtle populations on a global scale [11-13]. In particular, turtle strandings may also be considered a major threat to the populations of these animals in coastal areas [14].

Most importantly, according to Epperly *et al.*[15] and Cheng and Chen [16], the greatest impact on the survival of these animals is the use of fishing equipment, with emphasis on the fishing nets that are recognized as a major factor in the mortality of sea turtles across the world [17]. In Brazil, the homemade devices are commonly used on the coast [18]. Studies of the artisanal fisheries in Brazil are still few and there are no accurate statistics on such activity [19,20], representing a significant gap with regard to information on the bycatch of sea turtles [21].

Studies investigating the attitudes of members of traditional communities regarding the conservation of a particular resource can have great importance for the preservation of the ecosystem [22]. Furthermore, information derived from the community members’ knowledge of the environment can assist in management and co-management efforts, contribute to the existing knowledge of the biology of various organisms and their interactions with the environment [23] and provide important data to help shape the decisions of policy-makers and researchers [24,25].

Most of the interactions between humans and their environment are known to be mediated by feelings, behaviors, knowledge and beliefs [26]. Understanding and comprehension of the bio-cultural memory associated with the local knowledge of a particular traditional community as well as efforts to represent those community members and collaborate with their existence [27] are increasingly being utilized and incorporated in the responses to environmental and social changes [28].

The use of ethnoecological tools, like interviews and projective test, to evaluate possible damage and loss of biodiversity related to the populations of species under some degree of threat may represent a first step towards integrating the political management of natural resources and conservation strategies with the behavior of the local community so that fishery resources can be utilised rationally, with a consequent decrease in the mortality of sea turtles. From this perspective, this study investigates fishermen’s local ecological knowledge about sea turtles and attitudes towards the conservation and bycatch of sea turtles (Reptilia: Testudines) in Ilhéus, Southern Bahia, Brazil.

## Methods

### Study site

**Figure 1:**
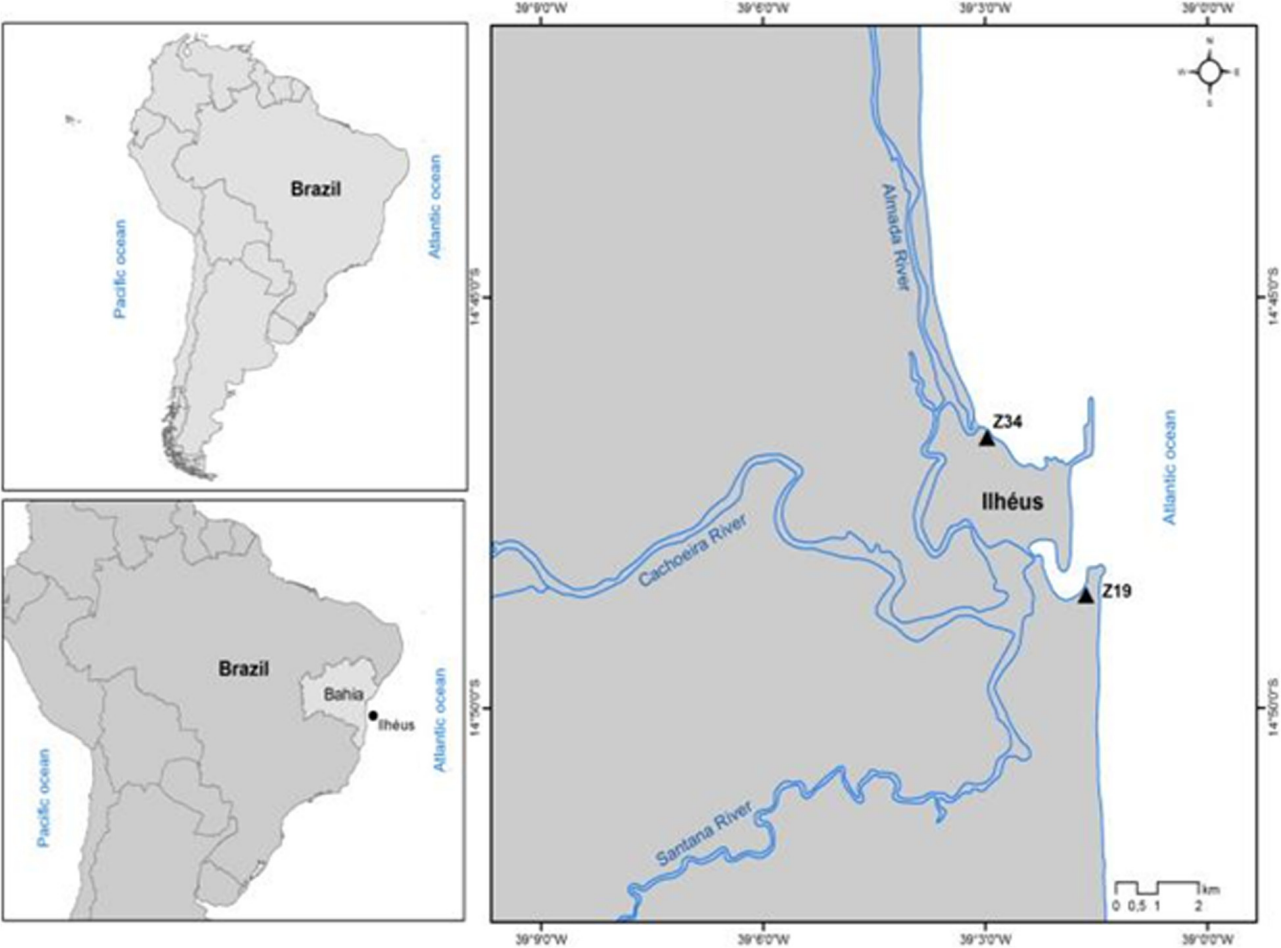
Locations of fishermen’s colonies Z-19 and Z-34, adjacent rivers and the continental shelf in Ilhéus, Bahia, Brazil.

The present study was conducted in the municipality of Ilhéus (14°48'40.44“S, 39°1'42.97”O; Figure 1), in the southern region of the state of Bahia, Brazil. This area has a population of 180,000 inhabitants and an area of 1841 km^2^[29]. The climate of the region, according to the Köppen classification scheme, is Af; warm and humid tropical, without a predictable dry season and with an average annual rainfall of 2000 mm [30].

The coastal region of Ilhéus is 80 km long and almost straight with few protrusions or recesses, bounded by the Sargi river to the north, the Acuípe river to the extreme south, and by the continental shelf, the edge of which passes between the 50 m and 60 m isobaths [31,32]. The hydrography of the region consists of two basins: the Cachoeira and the Almada. These basins are part of most of the routes of the fishermen of Ilhéus. There are two colonies of fisherman in Ilhéus: Z-19 and Z-34. These were chosen for use in this study because of the ease of finding fishermen to facilitate data collection. Located on the edge of Pontal Bay, colony Z-19 was founded in 1921 and is currently managed by José Leonardo Oliveira dos Santos. It comprises 3,000 members, of whom only 700 are active fishermen. Colony Z-34, located in the Malhado neighborhood, was founded in 1947, is currently managed by José Reynaldo Oliveira, and has approximately 3,000 active members, including individuals from neighboring municipalities [32]. These two colonies of fishermen are organs of the working class artisanal fisheries sector, legal form and with themselves in the city of Ilhéus, Bahia, Brazil.

### Procedures

Data collection took place from July 2010 to September 2011 in colonies Z-19 and Z-34. Interviews were conducted with experts fishermen. These fishermen were sampled using the method snowball method [33] in which fishermen in a particular locality indicate that people have greater knowledge and field experience among all fishermen. This sampling method was modified and adapted [34], where experts fishermen were selected through the initial indication by the president of the fishing colony. Field data were collected first through semi-structured interviews [35] and later through well-designed questionnaires which are suitable for research where it is desired to quantify the results subsequently [36]. The schedule of interviews to fishermen was adapted according to the forecasted arrival of the fishing boats, where we expected the best time for them to grant the interview. Constant contact with the research subject is observed as a necessary investment in studies of local ecological knowledge according to Brook [37].

**Figure 2:**
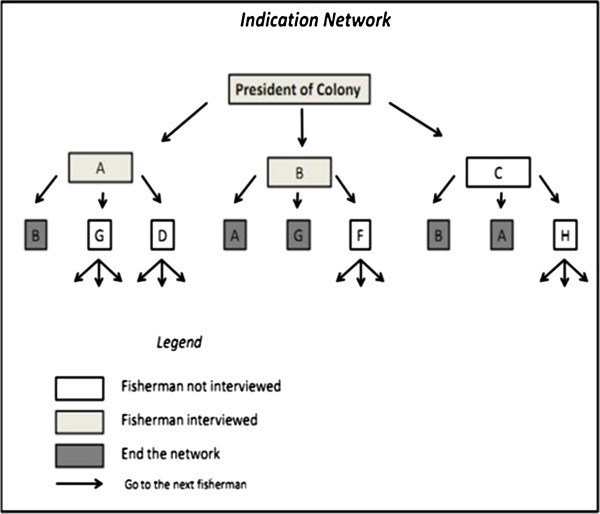
A model of the indication network used for the selection of expert fishermen in colonies Z-19 and Z-34 in Ilhéus, Bahia (N = 30).

Initially, the names of three fishermen who had relevant knowledge about fishing in Ilhéus were collected from the presidents of each colony using the criterion “native expert(s)”, meaning individuals who are self-acknowledged or recognized by the community as experts and persons that have a long history of fishing in the area [33,38,39]. The selected expert fishermen each indicated three additional fishermen and so forth, constituting an indication network (Figure 2). The network terminated when a fisherman was cited more than once. A fisherman was considered an expert if he was indicated two or more times. The fisherman experts were interviewed after being identified through the formation of the indication network. The interviews consisted of a series of questions (Table 1) relating to the fishermen’s profile, structure and work equipment, the local ecological knowledge (LEK) of fishermen about sea turtles and bycatch of sea turtles (It was considered local ecological knowledge of the fisherman on the ecology of the sea turtle and animal behavior), a projective test [40], attitudes towards turtle conservation (This part of the interview was assessed by the awareness of the respondent regarding the conservation status of turtles and their tendency to have positive action in relation to maintenance of the population of turtles) and beliefs and taboos regarding turtles (It was considered food preferences and aversions of fishermen and their implications for conservation of sea turtles). The projective test was performed by presenting six sea turtle species to record the perceptions and knowledge of respondents. To avoid skewing the test, the turtle *Lepidochelys kempii* (Kemps Ridley or Lora) was not used in the questionnaire due to its similarity to *Lepidochelys olivacea* (Olive), which is recorded in Brazil. Interviews were recorded on paper. We considered the common names of all species of sea turtles to assess knowledge through projective test. The questionnaire was pre-tested using a pilot study conducted in colony Z-18 in Itacaré, Bahia. Ten interviews were conducted in the pilot study. This procedure helped to adapt the questionnaire before applying it [41].

**Table 1:** Characteristics of questionnaire applied to the fishermen experts in Ilheus, Bahia, Brazil, 2010–2011

**Characteristics**	**Number of questions**	**Percentage of questionnaires**
Fisherman profile (sex, age, birthplace, nº of children, schooling)	10	12
Structure and work equipment (types of fishing boat, fishing activity)	19	23
Knowledge about turtles (feeding areas/nesting areas/dive time/ecological knowledge)	13	16
Projective test (identification of species of turtles through images/photos)	6	7
Knowledge bycatch (depth, capture location, likely species, state animal, fishing gear).	16	20
Attitudes towards conservation (probable reactions around turtles)	8	10
Beliefs and Taboos (eat, when, who can?)	10	12
Total	82	100

The fishermen were approached individually, with some interference from other fishermen present at the interview site. Despite this interference, the only responses recorded were those provided by the individual who was being interviewed. At the start of all interviews, the fishermen received a document entitled “Statement of Informed Consent (IC)” and agreed to participate in the research.

The interviews covered the emic approach with respect to the point of view of the research subjects [42]. The taboos were classified into the following categories (life history, temporal, habitat, specific, segmental, method) proposed by Colding and Folke [43] and the classification of fishing gear was based upon the “International Standard Statistical Classification of Fishing Gear” (ISSCFG) [44].

For quantitative comparisons of the respondents in terms of their broad knowledge and attitudes towards the conservation of turtles, indicators were created based on the study by Nazario and Bitencourt [45]. Data were converted using a three-point Likert scale for both knowledge (correct answers = 1, partial answers = 0.5, wrong answers = 0) and attitudes (positive attitudes = 1, moderate attitudes *= 0.5,* negative attitudes = 0). This scale quantifies the attitudes of individuals based on an order of numerical qualificative importance, expressing agreement or disagreement with respect to variables and attitudes related to the study object [46]. The indicators for local ecological knowledge and attitudes towards conservation were created by summing the scores for each subject and dividing the total by the highest possible score [45-47]. The reliability and internal consistency of these indicators was measured by Cronbach’s alpha coefficient, which assesses the magnitude to which the items in a group are correlated [48,49]. Knowledge and attitude indicators were divided into three classes (0–0.33; 0.34 - 0.66; 0.67 - 1). Attitudes were classified as positive, moderate and negative, whereas knowledge was classified as low, medium and high (based on Likert scale and Cronbach’s Alpha).

Correlation analyses were made between indicators of knowledge and attitude, investigating the relationship between profile variables of the respondent (time associated with the fishing colony, age and number of children) and knowledge and attitudes as well as the relationship between education level and knowledge and attitudes. We investigated the relationship between indicators and level of education by classifying education level as follows: A = illiterate; B = Elementary School 1 (1–5 years); C = Elementary School 2 (6–9 years) and D = Secondary school and Higher Education. Kruskal-Wallis (H) non-parametric tests, correlation analyses (r) and Cronbach’s alpha coefficient (α) were conducted using R version 2.12.1. The ltm package for R was used to calculate Cronbach’s alpha coefficient (α) [50].

## Results

### Profile of the fishermen

The indication network of colony Z-19 included 34 fishermen. Of these, 21 were considered experts. The network of colony Z-34 included 26 fishermen, of which only 13 were considered experts. Thirty experts were interviewed for the study. Only 4 experts refused to participate in the work.

The ages of the respondents ranged from 40 to 86 years, and they were all male. The average fishing experience was 32 years, and the majority of respondents had a low level of education (Table 2). We interviewed 7 illiterate fishermen and 3 who had completed high school. The time associated with the fishing colony varied from 3 to 52 years with a mean of 24 years, and 87% of respondents lived only on their fishing income. The predominant types of fishing boat in Ilhéus were fiber and wood, and fishing trips averaged 4 crew members. The boats were generally small (4–6 m width; 7–14 m length), 66% of the experts used the boats of other fishermen and the fishing gear most often used was fishing line and trawl nets. The average frequency of fishing trips was three to four times per month. The time at sea per fishing trip varied with the type of fishing gear used (trawl nets = 10–20 days; line = 7–8 days).

**Table 2:** Profile of the experts interviewed in the colonies of fishermen Ilhéus, Bahia (N = 30 – 100% male)

	**%**	**Minimum**	**Mean**	**Maximum**
Age (years)		40	54	86
Schooling (years)		0	5	14
Fishing time(years)		13	32	60
T associated the colony(years)		3	24	52
Time of residence in Ilhéus(years)		10	41	63
Nº of children		1	3	7
Occupation:				
Fishing only	87			
Others	13			

### Local ecological knowledge about sea turtles

The indicator of local ecological knowledge about sea turtles as measured by the Likert scale ranged from 0.26 to 0.77 with an average value of 0.43. In general, the local ecological knowledge about sea turtles was average. None of the candidates obtained the minimum or maximum values of the indicator. According to the established classes, 27% of the fishermen had a low level of knowledge about sea turtles, 63% had medium knowledge and 10% had a high level of knowledge. The Cronbach’s alpha coefficient for knowledge was approximately 0.7. The index of knowledge was not associated with the age of the fishermen (r = 0.10, p = 0.62), the number of children of the fishermen (r = 0.08, p = 0.66) or the time associated with the fishing colony (r = −0.05, p = 0.80).

**Figure 3:**
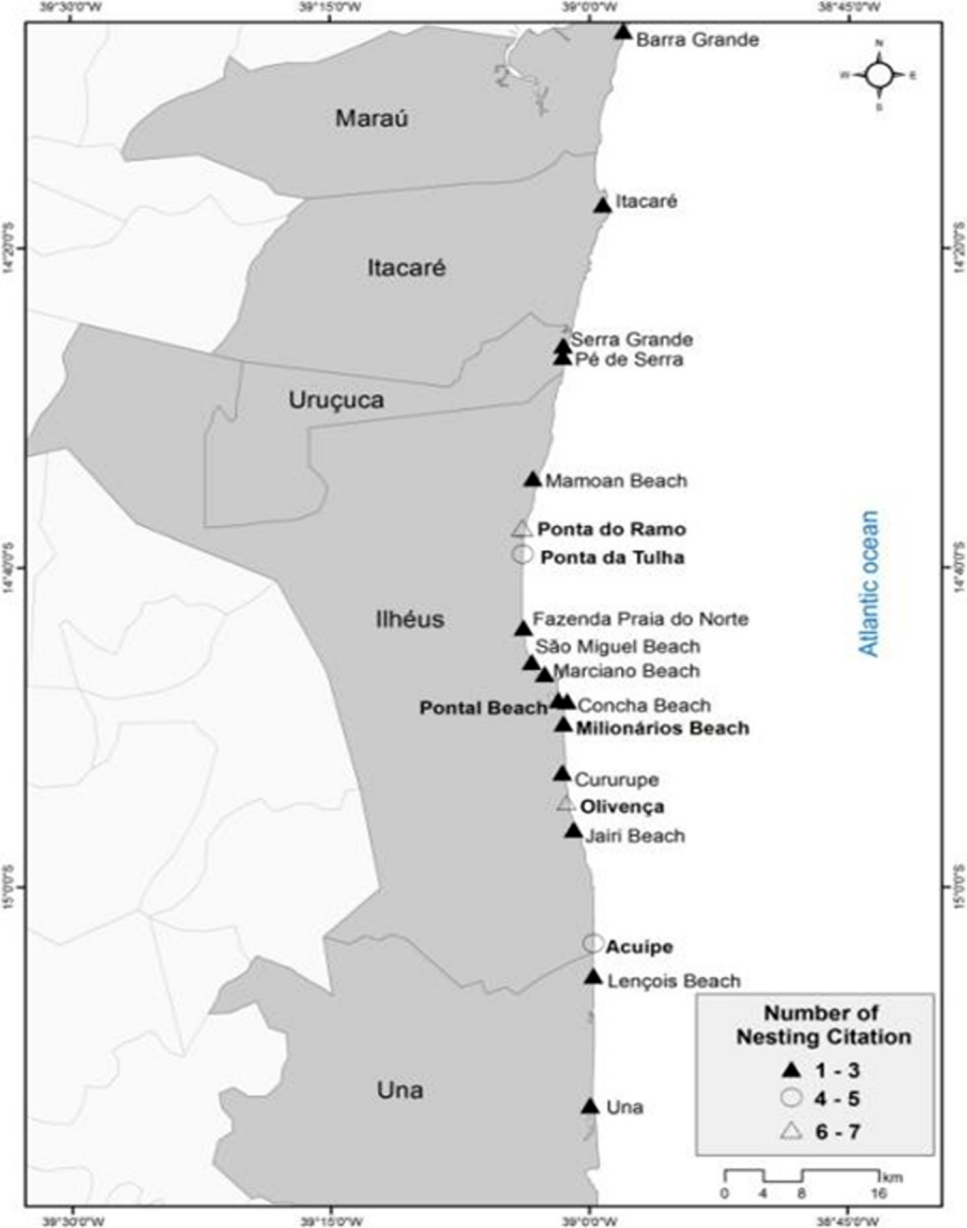
Nesting areas in southern Bahia according to the interviewed experts (N = 30).

All fishermen said they had seen turtles along the coast of Ilhéus. The experts cited 20 nesting areas from Barra Grande to Una covering the entire coast of Ilhéus. Of these, Olivença and Ponta do Ramo were most frequently cited by experts as nesting areas for sea turtles (6–7 times). Ponta da Tulha and Acuípe were also remembered as spawning areas (4–5 times) and the remainder were cited by at least one respondent (1–3 times, Figure 3). In relation to nesting areas, all fishermen said that turtles spawn on desert beaches, and 38% said that spawning occurs mostly in the summer.

In the projective test, only one fisherman mentioned that *Natator depressus* occurs in the region. All others cited at least one species that occurs on the coast of Bahia. Of the experts interviewed, 45% correctly identified the species *E. imbricata;* 24%, *C. mydas*; 10%, *C. caretta;* (being all respondents belonging to the classes of medium or high knowledge) and 13%, *D. coriacea* (only one respondent belonged to the class of low knowledge and the rest belonging to the other two classes). No fisherman identified the species *L. olivacea*. Most experts identified the turtles based on their shell, colour, size and fins. It is important to note the record of *D. coriacea* made by a fisherman interviewed in the south of Ilhéus, specifically on the high seas in front of Comandatuba Island. This fisherman was one of the four respondents who recognized the leatherback turtle and described some details consistent with the literature.

The interviewed experts identified the various types of turtle by their common names, referring to *C. mydas* as the green turtle, *suranha* and *aruanã*. The species *E. imbricata* was identified as the hawksbill turtle and as *malhada*. *C. caretta* was identified as the common and the yellow turtle*. D. coriacea* was identified as the leathery, skin, and black turtle and as *Jamanta. L. olivacea* and *N. depressus* were not identified by the fishermen by their common names.

When asked to identify turtle foods, all experts cited at least one type of food correctly according to literature [51-53] and when asked about the predators of turtles, they cited men, sharks, some birds (albatrosses and black vultures) and fish (goliath grouper, common dolphinfish and shark). Responses provided regarding the turtles’ dive time varied significantly between respondents and 53% mentioned a correct time interval with reference to some studies [54-56]. According to the fishermen, the preferred habitats of the turtles are places with rocks, reefs, beaches and shallow and deep water. The fishermen reported that the turtles’ diet consists of seaweed, small fish, crustaceans, limestone, shellfish and shrimp. Plastics and several wastes were also cited as parts of their diet. Although the overall level of knowledge was medium, some fishermen showed satisfactory knowledge compared with the literature on the ecology and behavior with regard to the chelonians studied.

### Knowledge about bycatch

Only 1 expert had never accidentally caught a turtle during a fishing operation. During each respondent’s last reported capture, 66% were using fishing line as their fishing gear and 94% of the captured turtles were alive and in a normal state without apparent injury. Their average weight was 31 kg and depth was 36 m at the last sighting. The coasts of Ilhéus, Olivença and Acuípe were cited as the localities with the highest numbers of records for turtle captures. In the interviews, most of the fishermen attributed their most recent turtle capture to fishing line, but all experts said that the fishing gear that picks up the most turtles in the region of Ilhéus is the nets (gill/lobster, n = 27; shrimp trawl, n = 2) and that turtles are rarely captured by lines (fund, submerged or half water; n = 1) or long-lines (n = 1).

### Attitudes towards conservation

The indicator for attitudes towards conservation of sea turtles had an average value of 0.69. The study participants proved to be alerted before the causes of the decline of sea turtle population, tending to present awareness and actions more accurate than negative that can assist in the recovery of the conservation status of the species studied. This ranged from 0.35 to 1 and only 2 fishermen obtained the maximum value. Most of the interviewees (59%) had positive attitudes the maintenance of the sea turtles population in the study region and the other 41% held moderate attitudes. No negative attitudes were recorded. The Cronbach’s alpha index calculated for attitudes was 0.43. The index of attitudes was not correlated with time associated with the fishing colony (r = −0.18, p = 0.35), the respondent’s age (r = −0.28, p-value = 0.15) or the respondent’s number of children (r = −0.04, p = 0.83).

Ninety percent of respondents thought it was important to conserve turtles and the environment where they live. Ninety-seven percent did not think that sea turtles affect fishing, but only 47% knew how to explain this fact. Only one interviewee did not approve of the law that has prohibited the capture and use of sea turtles in Brazil since 1986 (Decree of SUDEPE, paragraph 005 of January 31, 1986, IBAMA 2009) along with other complementary legislation. Seventy-three percent of the experts had previously held unfavorable attitudes related to the consumption of sea turtle eggs, but all said that they no longer consumed the eggs in the present.

In a hypothetical encounter with a turtle, 3 fishermen said that they would consume it or use the shell for making hand-crafted products. When asked about how to avoid catching turtles, 27% said that they did not know how and 73% said that they avoid using fishing nets (shrimp and lobster) to avoid catching turtles catch.

### Local ecological knowledge and attitudes towards conservation

**Figure 4:**
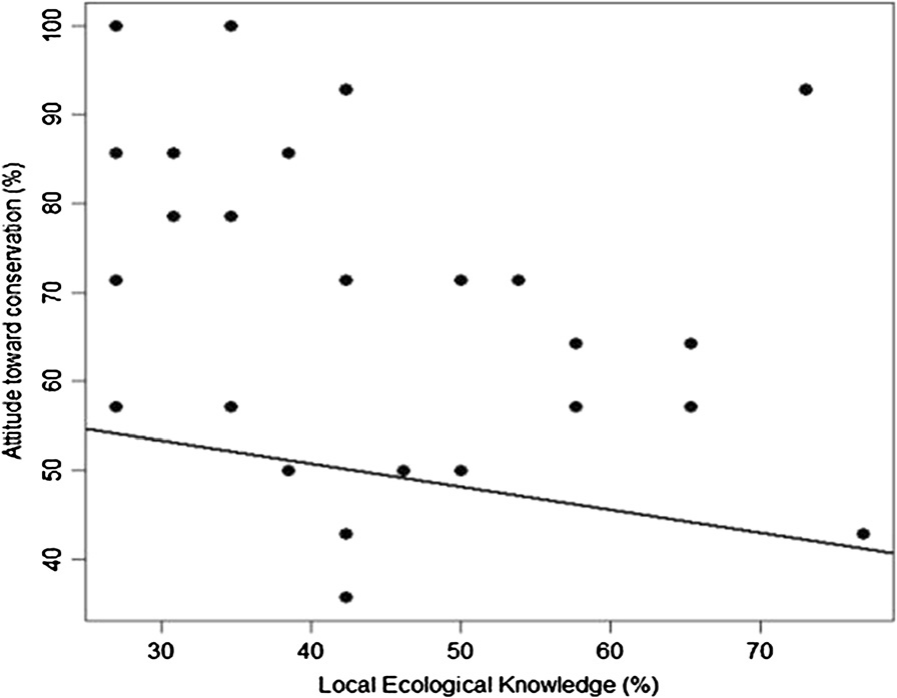
The correlation between local ecological knowledge about sea turtles and attitudes towards their conservation (p = 0.04, N = 30).

In the present work, there was a tendency for attitudes towards turtles to be inversely related to knowledge about sea turtles. The correlation between these two indicators was negative and significant (r = −0.38, p = 0.04). The interviewed experts who exhibited a greater local ecological knowledge about sea turtles tended to have more unfavorable attitudes towards the conservation of chelonians (Figure 4).

### Relationship between level of education and the indicators

**Figure 5:**
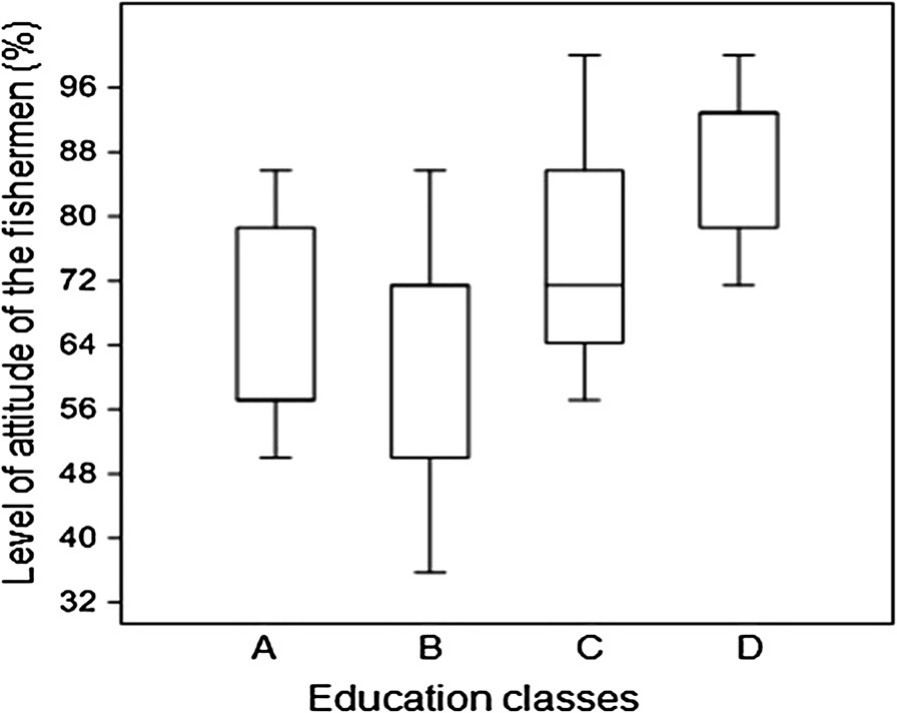
The correlation between the level of favourable attitudes of the fisherman towards conservation (%) and education categories (A = illiterate, B = Elementary School 1, C = Elementary School 2, D = Secondary school and higher, N = 30).

In this study, the fishermen’s level of education did not influence the extent of their local ecological knowledge about sea turtles (H = 1.27; p = 1.74). Regarding attitudes towards conservation, there was a tendency for more highly educated fishermen to have more positive attitudes towards turtle conservation (H = 8.33; p = 0.04; Figure 5).

### Beliefs and taboos

In the study of the perceptions of the fishing communities of Ilhéus, sea turtles were mentioned as taboo by 48% of the interviewed experts. They were considered taboo as food due to their “strong” meat, for presenting leathery shell, and have the ability to cause disease when ingested. The turtles were locally referred to as “*remoso”* and “*carregado*”. The folk medicine of some of the fishermen included uses for sea turtles (21%). Therapeutic indications for human use that were mentioned by the experts included the use of turtle lard oil as a remedy for rheumatism, muscle aches, fatigue and back pain and for fighting bronchitis and asthma.

Specific taboos were recorded for situations in which individuals had dietary restrictions defined in relation to sea turtles. The situations comprised the following: post-operative periods for both sexes, some types of inflammation, chronic disease and pregnancy in women. Of the interviewed experts, 4 said that they did not consume turtles with external characteristics such as the presence of warts and lumps on the body of the animal or the presence of a jellyfish on the face. The justification was that consuming turtles with these characteristics may harm a person’s health.

Life history taboos were also observed, in which 45% of respondents said that they only eat adult and elderly turtles and restrict their use of the species at early stages of its development (young). Habitat taboos were also recorded for 24% of respondents. According to the interviewees, there are places where fishing access is limited due to the presence of turtles. Fishermen cited the following locations: Pé de Serra, Ponta do Espigão, Pedra de Ilhéus and Abrolhos as places where they avoid fishing because of the large number of turtles found in these areas.

Among the 4 experts who said they have used the shells of sea turtles, primarily hawksbill turtles, to make glasses and other handcrafts, there was no food restrictions related to the consumption of eggs. The fishermen do not use the meat for commercial purposes. When a turtle is captured, they consume it at sea or take the meat home. Fifty-two percent of the respondents enjoy turtle meat compared to beef and chicken.

## Discussion

### Knowledge about sea turtles

Keeping in mind that the survey was conducted only with fishermen experts who had been recognized as knowledgeable about fishing in the region, one can say that we recorded as much information as possible and that no other local fishermen had different or deeper knowledge than what we recorded. The index we generated to measure local ecological knowledge presented an acceptable degree of reliability. According to Gabriel and Tritapepe [57], values of Cronbach’s alpha above 0.6 are considered satisfactory for opinion polls. The local ecological knowledge about sea turtles according to the Likert scale was predominantly medium and high, which was expected due to the long fishing trips, which helps to increase the probability of encounters with animals. Regarding indicator individually, no fisherman got full or zero knowledge about the animal, which was expected, because most fishermen have already reported some contact with turtle at sea or on the beaches, which helps to generate information habitat, breeding and feeding sites and nesting.

It was assumed that the fishermen who were identified by the indication networks in the two colonies had a deeper knowledge of turtles. This assumption may be incorrect considering that fishing effort is directed towards species of fish and laws are currently in place in Brazil that restrict fishing for turtles, with the result that fishermen’s contact with turtles is only casual. Most of the fishermen experts we interviewed also use a bottom line, which is considered a type of fishing gear that is less likely to catch turtles compared with other types of gear [58]. Calo *et al.*[32] concluded that the fishermen of the same study area possess considerable knowledge about snapper fish (Actinopterygii: Teleostei) “vermelhos”, a group of large fish, knowledge which was possibly acquired by exploiting the fish with fishing gear, an activity which does not occur with the sea turtle.

Sea turtles are considered key species in coral reef communities [53,59]. Many interviewed fishermen reported avoiding fishing in areas where there is a greater probability of interaction with the animal. Decree No. 037 regulating Municipal Law No. 3212 of 01/30/2006 was completed in 2011 for the realization of the creation of Municipal Marine Park Ilhéus to protect some marine species, with an emphasis on *Epinephelus itajara* (the goliath grouper). Even while under construction, the existence of this initiative may explain the avoidance of this type of ecosystem by fishermen in the region, which in the future may have a positive effect on the population growth of sea turtles in the region. However, the regulations imposed on fishermen to reduce fishing should always be constantly monitored and revised because the survival of individuals in fishing areas may often depend on the attitudes of the fisherman, who in a given time can be influenced negatively, especially when there is an initial assessment appropriate to the likely impacts to coastal communities [17]. Another reason for the low capture rate of turtles is that contact between fishermen and the animal may cause a decrease in fishing effort, generating financial and material losses, even if minimal. Marcovaldi *et al.*[60] report that fishing-animal interactions can cause damage to the fishing target. Nevertheless, the fact that many interviewees said that turtles do not affect their fishing is not for the simple reason that turtles do not cause any equipment damage or reduction in effective fishing time, but because they have only occasional contact with turtles and consequently the probability of damage caused by turtles is low. This fact can also be a reflection of the continued work of the Tamar as in many parts of the Brazilian coast, through which many anglers may have absorbed a more conservationist discourse that is not necessarily (but can be) a reflection of realities in their day-to-day.

Despite the finding that the fishermen’s local ecological knowledge about sea turtles as communicated through interviews was not within the range that represents deep knowledge, the interviews with experts did identify areas of great importance for nesting turtles. In these sub-areas of Ilhéus on the southern coast of Bahia, there are records of strandings of four species of sea turtles [14]. The increased recognition of hawksbill turtles in the projective test can be explained by the fact that hawksbill species have been recorded nesting on the southern coast of Bahia [61] relation to the projective still some limitations methodology as shown figures (drawings and pictures two-dimensional), the color variation of the hull according to the animal immersion in water may have been implicated in the recognition species.

In the municipalities of Itacaré and Uruçuca, there are reports of turtle nests on the beaches of Pompilho, Itacarezinho and Patizeiro [62]. Due to the hawksbill turtle’s peculiarities, for decades products have been extracted from the animals for export and sale to tourists [63]. Among all species, the hawksbill turtle is the one that has suffered the greater depredation as a result of its shell [64]. The hawksbill turtle’s shell holds more use than those of other species, and their greater contact with fishermen may have influenced the collaboration and greater recognition on the part of respondents.

There are few studies in the literature that address the history of the leatherback turtle in the state of Bahia. The current conservation status of the species is “in critical condition” [3,5]. There are sporadic reports of spawning in the extreme south of Bahia [65], and the shore in the state of Espírito do Santo is cited as the most important nesting area for this species in Brazil [66], with a population that is genetically differentiated from the rest of the country [67]. In the present study, some fishermen were able to identify the leatherback turtle, even if they had only fished in southern Bahia throughout their lives. Huntington [36] emphasizes the importance of incorporating this type of local ecological knowledge into research projects and management strategies and of integrating, analyzing and incorporating this new knowledge. Thus, this type of initial information can be an important step for the conservation of potential new nesting areas of the species in southern Bahia, where studies on population structure and nest monitoring are nonexistent.

Even with a few catches to the bottom line in the study area, fishermen warned of the impact of the use of net fishing in the region. Trawling for lobster and the use of fishing nets have been identified as one of the main threats to sea turtle populations worldwide [12,17,68,69]. In Brazil, the impact of lobster trawling in Bahia has already been observed [70]. These methods can drive large decreases in sea turtle populations because forced apnea may further aggravate the state of captured turtles, which can lead to death [71].

*Attitudes towards conservation and their relationship with other variables* - The low Cronbach’s alpha value suggests that the items analyzed express different attributes and cannot be jointly adopted in the calculation of a one-dimensional variable [45]. For Pereira [58], there is a stipulated amount of alpha needed to determine the validity of an indicator. Values above 0.40 have been satisfactory for some studies [47,72].

Fishermen experts demonstrated predominantly positive attitudes. Coastal communities with nesting areas in Sri Lanka exhibited similar attitudes [73]. The influence of community attitudes towards conservation in traditional and some demographic variables has been almost nonexistent [74,75]. Negative attitudes were provided by fishermen with a greater number of children because it is expected that a family with a large number of members requires a greater amount of food energy for their sustenance. In Boer and Baquete [76] also found no relationship between number of children and attitudes around an elephant reserve in Mozambique. Even with environmental education activities and occasional lectures given to members of the local community, no trend was identified between attitudes and time associated with the fishing colony. As was observed in the fishing community in this study, the age of the respondents did not influence attitudes in two protected areas in Nepal [77]. Mehta and Keller [78] also documented the same trend. Thus, the profile variables of the respondents recorded in this study did not influence the attitudes of the respondents in relation to the conservation of sea turtles in the region of Ilhéus.

More unfavorable attitudes towards the conservation of sea turtles were held by those fishermen who know more about their behavior and could best distinguish their habitat characteristics. It is expected that foraging and capture of turtles are easier for those fishermen who have a more enhanced level of knowledge about the resource. Bright and Tarrant [79] reported that knowledge increases the ability to think, looking at all sides of the issue, but does not always influence the direction of the attitudes of an individual. In this case, the above knowledge about the rules of endangered species in the United States did not influence the perceptions and attitudes of students.

These unwritten social rules can be a way to conserve a resource. Fiallo and Jacobson [80] found the same relationship between these two variables. Positive attitudes were exhibited by those with a higher level of education, perhaps because the access to the information acquired during their studies, access to different types of media and greater contact with educated people could help in better assimilating the importance and need for conservation of resources that are threatened with depletion. According to Sah and Heinen [81], attitudes towards the conservation of a resource are influenced by educational level. However, there were some fishermen with low education levels who had positive attitudes towards the conservation of sea turtles. The fear of fines and punishments meted to those who violate environmental laws [47] may help explain this finding. The attitudes of these fishermen may also be influenced by some kind local belief. Bright and Barro [82] showed that beliefs can have influence over the attitudes of an individual in relation to natural resources in addition to just their knowledge.

### Beliefs and taboos

Food taboos may be considered informal institutions that define and limit the use of resources by human communities in ecosystems, accounting for rules that are not instituted but somehow regulate human behavior [83]. In this study, the presence of food taboos can be considered a reason for the low consumption of sea turtles by fishermen of the macro-region of Ilhéus. These unwritten social rules can be a way to conserve a resource [23,70]. However, a decrease in adherence to traditional practices over time can cause a greater impact on some populations of animals and plants [84].

The specific taboos that were identified by interviewing experts of the two fishing colonies of Ilhéus are similar to those reported in studies from the Atlantic Forest and the Amazon [85,86]. Decreases in the exploitation of wild species can be aided by specific taboos [43]. Dietary restrictions related to the appearance and taste of meat was considered more a means of avoiding the consumption of the animal. The reasons attributed to the taboos by the fishermen were the same as those given by other fishermen in coastal communities in the southeast [85].

Exogenous taboos, in which laws are imposed on the population leading to a breakdown of the interaction between people and animals [87], may in some cases assist in the conservation of a resource. Often, this kind of taboo cannot control all of the actions of the fishermen, as occurs in the fishing community of Ilhéus. The chelonians, especially sea turtles and turtles, are one of the most popular ingredients used in traditional medicine around the world [88]. In northeastern Brazil there are several records of the use of animals and plants in alternative therapies [89].

In Bahia, turtles are known to be highly utilized in zootherapy and alternative medicine [90] as well as in communities in the southeast of São Paulo [85,91] and in Rio Tocantins [86]. In the region of this study as well as in other traditional communities, turtle fat is used for the treatment of asthma, bronchitis and arthritis [85,92]. On the north coast of Bahia as well as on the coast of Ilhéus in southern Bahia, fat is only used in this way when there is bycatch [87].

### Conservation concerns

Among the species of sea turtles that are recorded in Brazil, all are under some degree of threat [3-7]. The use of bycatch in nets by traditional coastal communities is a major factor in the declining populations of turtles [93].

In relation to fisheries, lobster and shrimp trawling should be adapted and restricted in certain ecosystems due to the high probability of turtle capture. Fishing colonies should always be involved in any change in fishing regulations, and participation in key decisions should be interactive. Despite the fact that this type of fishing is not considered a major contributor to the problem of bycatch worldwide, this type of fishing should still be constantly monitored due to its large magnitude and its ability to generate deep local environmental effects [94].

Data acquired from local ecological knowledge can assist in strategies and programs for sustainable conservation and management policies [24] and generate important discussions to strengthen the understanding of the resource under study [95]. The preservation of traditional practices such as sustainable food taboos, beliefs and customs is necessary. In relation to the sustainable use of resources in zootherapies, there must be a local analysis and a thorough observation of the possible implications of the practice, as well as a check for immediate needs to provide appropriate management measures as it relates to the conservation of the species [89].

An individual with a higher knowledge rating did not have more positive attitudes towards turtles in our study. However, mitigation measures related to environmental education should not be discarded, but should be reviewed and explored in a more appropriate way. Recommendations are needed for effective communication strategies in cases where there is a propensity towards negative attitudes by fishermen with a greater degree of knowledge about the ecology of the animal. Even in the generally positive attitudes that prevail among the interviewed experts, there are still fishermen who occasionally use turtles which can be detrimental to the species, especially females who are more coastal and more likely to be predation.

Studies monitoring the nests, clutches and local ecological knowledge of sea turtles in southern Bahia are needed due to the scarcity of data in the existing scientific literature, especially for the leatherback and hawksbill turtles, which need urgent help because of their critical conservation status. Strategic conservation and mitigation measures must be developed and applied so that a significant portion of the existing diversity of turtles is not lost over the coming centuries [96].

The involvement of the community in conservation activities and monitoring may, over the long term, contributes to increased knowledge and more favorable attitudes [97]. Promoting beliefs and taboos conducive to sea turtles conservation could foster positive attitudes and behavior.

## Conclusions

Due to the series of threats that this population group is confronting animal comes into the world, ethnoecological studies are recommended for areas where gaps exist regarding the population structure and conservation status of marine turtles as identified in this study.

According to experts fishermen in Southern Bahia, the incidental capture of sea turtles is occurring, these being mainly attributed to fishing line, because most fishers use this fishing gear with main instrument of fishing. However, respondents acknowledged the fishing nets as major factor of mortality of sea turtles in southern Bahia.

Traditional knowledge about the biology of the animal was evidenced, analyzed and compared with the scientific literature on zoology and biology of the animal was a series of confirmations of such knowledge.

## Competing interests

The authors declare that they have no competing interests.

## Authors’ contributions

HOB and AS - Writing of the manuscript, literature survey and interpretation; HOB – Collected, organized and analysis data; AS - Contributed with ideas on the study and discussion, involved in revising the manuscript. All authors read and approved the final manuscript.
